# Myocardial Infarction as the First Clinical Manifestation of Coronary Artery Disease: A Scoping Review

**DOI:** 10.3390/jcm15072603

**Published:** 2026-03-29

**Authors:** Maya D’Angelo, Angeliki Psycharis, Nicolo Piazza, Giuseppe De Luca, Elvin Kedhi

**Affiliations:** 1 Department of Medicine, McGill University Health Centre, Montreal, QC H4A 3J1, Canada; maya.dangelo@mail.mcgill.ca (M.D.); angeliki.psycharis@mail.mcgill.ca (A.P.); 2 Centre for Outcomes Research and Evaluation, The Research Institute of McGill University Health Centre, Montreal, QC H4A 3J1, Canada; nicolo.piazza@mcgill.ca; 3 Division of Cardiology, McGill University Health Centre, Montreal, QC H4A 3J1, Canada; 4 Service of Cardiology, AOU “Policlinico G. Martino”, University of Messina, 98121 Messina, Italy

**Keywords:** myocardial infarction, prevention, risk stratification, asymptomatic, coronary artery disease

## Abstract

**Background/Objectives**: Acute myocardial infarction (AMI) remains a leading cause of death worldwide and sometimes occurs as the inaugural presentation of CAD. Studies have been heterogeneous in reporting what proportion this population represents; therefore, we sought to review the evidence of myocardial infarction as the initial manifestation of CAD. **Methods**: We conducted a scoping review of 25 studies (1979–2021) assessing the prevalence, risk factors, and outcomes of patients who experience AMI as the first clinical manifestation of CAD. **Results**: Across studies, most found that half of AMI patients present with no prior angina or CAD diagnosis. These patients tend to be younger and have fewer traditional risk factors. Sex differences were inconsistently reported, though some studies suggest that women may be more likely to present with unheralded AMI. Diabetes and hypertension were interestingly more common in patients with known CAD. Patients with unheralded AMI demonstrate a larger infarct size and may have a higher likelihood of adverse cardiovascular events compared to those with known CAD. **Conclusions**: Our findings highlight a critical gap in the current risk models of CAD evaluation, which are often symptom-based and focused on detecting ischemia, thus failing to detect a significant proportion that present with AMI as their initial manifestation of CAD.

## 1. Introduction

Acute myocardial infarction is the most severe presentation of coronary artery disease. Unfortunately, it can also be the first clinical manifestation of this disease [1,2]. In other words, asymptomatic individuals may go unrecognized until a major cardiac event occurs. AMI occurs when there is a rupture or erosion of atherosclerotic coronary plaque. A thrombus that occludes the vessel will typically lead to an ST-elevation myocardial infarction (STEMI). On the other hand, partial occlusion or total occlusion in an area supplied by a collateral circulation can result in non-ST-elevation myocardial infarction (NSTEMI) or unstable angina [3].

Coronary artery disease (CAD) remains the leading cause of death globally [4], and according to the World Health Organization, accounts for 13% of total deaths worldwide [5]. It represents a significant burden on healthcare systems, and is a major target for many primary and secondary prevention initiatives [4]. While the classical symptom of CAD is exertional chest pain, often referred to as stable angina [6], its clinical presentation can be heterogeneous. For instance, some patients experience anginal equivalents such as dyspnea, nausea, or fatigue [7], and others may remain completely asymptomatic despite significant underlying coronary disease [8]. Indeed, the Miami Heart Study showed that in a cohort of 2359 asymptomatic individuals (mean age of 53 years), 49% had plaque on their coronary computed tomography angiography (CCTA), with 6% having coronary stenosis >50% [9].

The phenomenon of an acute myocardial infarction (AMI) as the first clinical manifestation of coronary artery disease (CAD) raises important clinical and epidemiological questions. Do these individuals represent a distinct subgroup with unique or underrecognized risk factors? And if so, should this raise questions about current screening and risk stratification guidelines? Standardized risk scores, like the Framingham score, are commonly used to predict first coronary events [10]; however, they may fail to identify individuals who ultimately experience AMI without any prior diagnosis of CAD. It is unclear whether this specific patient population differs from those with symptomatic or previously diagnosed CAD in terms of risk profiles, presentation, and outcomes. While several studies have attempted to characterize this population, the existing literature remains limited and heterogeneous. By understanding available evidence, this review aims to better understand why these individuals go unrecognized and whether improved CAD screening and diagnosis could lead to better outcomes.

This review aims to synthesize available evidence on this topic, with a focus on the reported incidence of AMI in patients without prior known CAD, the associated patient demographics and characteristics, biochemical and imaging findings, angiographic features, and clinical outcomes.

## 2. Materials and Methods

### 2.1. Study Design and Scope

A scoping review was conducted to collect the available evidence regarding patients who present with acute myocardial infarction as their first clinical manifestation of CAD. This methodology was selected as there are a limited number of studies addressing this subject, there is significant heterogeneity in study designs, diverse definitions of what is considered a “first presentation” or “known CAD” and temporal shifts in screening, diagnosis and treatment. No formal protocol was registered for this scoping review.

### 2.2. Search Strategy and Data Sources

**Figure 1:**
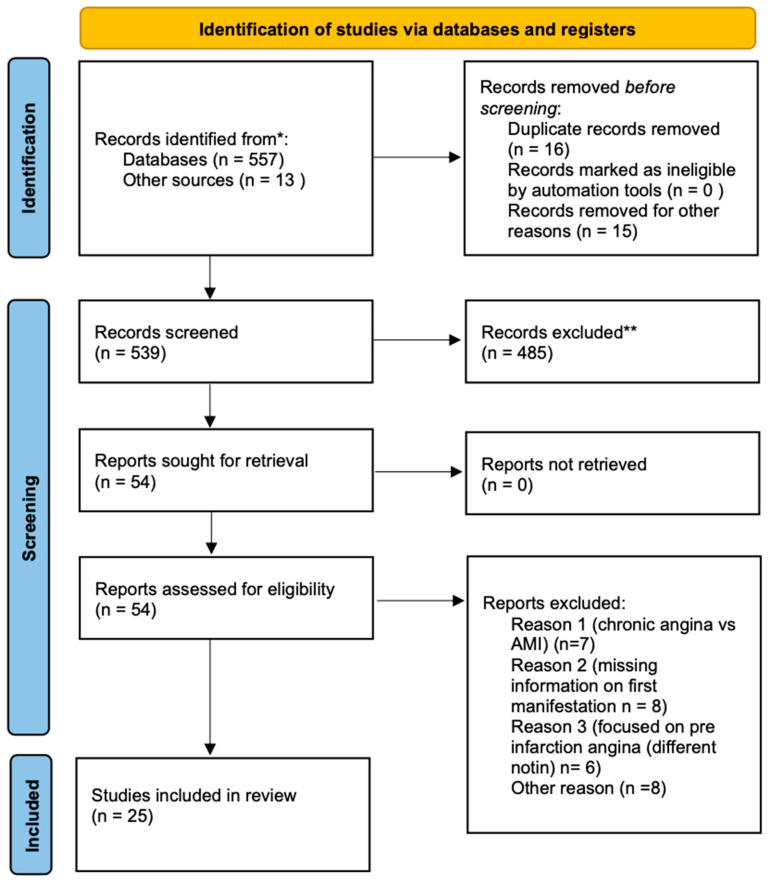
PRISMA-ScR flow diagram of the study selection process. * Other sources described in Section 2.2, ** Exclusions based on title screening.

An electronic literature search was performed in PubMed/MEDLINE [1970–2024] in February 2025 from which we derived the PRISMA-ScR (Preferred Reporting Items for Systematic reviews and Meta-Analyses extension for Scoping Reviews) flow diagram (Figure 1). We used a combination of controlled vocabulary (MeSH) and free-text keywords. The search strategy included terms for acute myocardial infarction (e.g., Myocardial Infarction [MeSH], myocardial infarction, acute myocardial infarction), angina (e.g., Angina Pectoris [MeSH], angina), and terms indicating angina occurring prior to infarction (preceding, preinfarction/pre-infarction, antecedent, prior/previous, before, prior to), as well as terms suggesting an initial presentation (first, first time, initial). The PubMed search returned 557 records that were taken forward for title/abstract screening (Figure 1). To ensure a comprehensive capture of the relevant literature despite heterogeneous indexing terms (e.g., ‘unheralded MI’, ‘preceding angina’, and ‘previously known CAD’), the electronic database search was supplemented by backward and forward citation screening (citation-chasing) of all included studies using Google Scholar/Cochrane/PubMed. Relevant peer-reviewed conference abstracts were included; when subgroup data was available only in abstract form, the corresponding full-text registry publication was used to understand the population studied and methodology.

### 2.3. Eligibility Criteria and Study Selection

Studies were included if they reported on patients presenting with AMI, including NSTEMI and STEMI, as their inaugural presentation of CAD and if they had information on prior angina or prior diagnosis of CAD. Prior angina was defined as symptoms > 1 month before the event. Notably, studies on only “pre infarction angina” in the days leading up to AMI were excluded. Studies comparing patients with chronic angina vs. AMI were excluded, as the primary focus of this review was restricted to patients presenting with AMI, among whom the presence or absence of prior CAD history was assessed. Given the timelapse between studies and advances in CAD screening and detection, we divided studies into two categories: (1) symptom-based (majority of studies): studies defining the first presentation as the absence of prior angina; and (2) diagnosis-based: studies defining first presentation as the absence of prior documented diagnosis of CAD.

### 2.4. Data Extraction and Synthesis

A standardized, multi-stage screening process was conducted: initial titles and abstracts were screened for relevance, followed by a full-text review of candidate articles by the study team. Data from 25 studies were extracted in an Excel document, including country, population, definition of first manifestation, main prevalence findings, key clinical characteristics and outcomes reported. Data were charted by a single reviewer using a standardized charting form. Any uncertainties were resolved through discussion with the senior author. Given the inherent secular bias and significant heterogeneity in study populations and diagnostic criteria, a qualitative synthesis was performed. Results are presented as a narrative overview supplemented by a PRISMA-ScR flow diagram to document the selection process. In accordance with scoping review methodology (PRISMA-ScR), a formal critical appraisal of the individual sources of evidence was not performed.

## 3. Results

### 3.1. Prevalence of AMI as the First Presentation of CAD

A total of 25 studies published between 1979 and 2020 were included in this review. The majority of studies utilized retrospective or prospective cohort designs, with sample sizes ranging from 31 to over 120,000 participants. The characteristics, definitions of prior CAD, and primary findings of each study are detailed in Table 1. Studies between 1979 and 2002 reported that approximately 50% of patients with AMI had no prior history of angina >1 month prior to AMI [11,12,13,14,15,16,17,18]. For instance, in a 1979 retrospective study, Harper et al. reported that 48% of 577 AMI patients had no prior angina [16]. Behar et al. (1992) found that 57% of 4166 patients with first AMI had no preceding angina [11]. A few of these earlier studies suggest that the proportion of patients experiencing angina prior to AMI is approximately a third [19,20,21,22,23,24,25], while others suggest this proportion may be even less [26,27,28,29,30], meaning the majority of these patients would have experienced an “unheralded AMI”.

More recent large-scale studies have transitioned from looking into the presence of prior angina to the presence of diagnosed CAD or atherosclerotic cardiovascular disease (ASCVD), which may include peripheral arterial disease or cerebrovascular disease. In a 2018 French registry, only 26% of 13,130 AMI patients had known CAD. This translates to 74% of patients with AMI who have never been diagnosed with CAD before this event. Furthermore, in this same study, the proportion of patients with AMI as the first manifestation of CAD increased significantly from 2005 to 2015 [31,32]. Similarly, Siudak et al. analyzed the largest cohort to date, which consisted of over 120,000 patients from a Polish registry, and found that 77% of AMI cases occurred in patients without prior CAD [1,2].

It is difficult to compare epidemiological trends given the different categories of studies, one being symptom-based and one being diagnosis-based. However, on of the more recent studies in 2014 pooled these two groups: they defined ischemic presentation as atherosclerotic disease on electronic health records or the clinical presentation of chest pain. Out of their cohort of 16,439 patients with AMI (STEMI and NSTEMI), 8364 (51%) had no prior ischemic presentations [33]. A similar study looking at the presence of ASCVD in patients with AMI had STEMI and NSTEMI subgroups and showed that only 29% of STEMI patients had known ASCVD whereas 50% of NSTEMI patients had ASCVD [34]. Similarly, the Framingham study showed that 68.5% of men and 60.5% of women had no pre-existing cardiovascular condition before AMI, including a history of angina [35].

**Table 1:** Characteristics of included studies.

Study (Year)	Design	Population (N)	Definition of Prior Angina/CAD	Main Prevalence Finding	Key Outcomes Reported
Anzai et al. (1995) [20]	Prospective cohort	291 (First Q-wave AMI)	Chronic angina (>1 month before AMI)	25% and 29% had chronic angina before anterior and inferior AMI, respectively	Lower peak CK levels in patients with any history of angina
Barbash et al. (1992) [26]	Prospective trial cohort (secondary analysis)	8329 (AMI with thrombolysis)	Antecedent angina (>1 mo vs. <1 mo)	47% had preceding angina	Evaluated outcomes in the context of thrombolytic therapy
Behar et al. (1992) [11]	Retrospective cohort	4166 (First AMI)	Chronic angina ≥1 month before onset of MI	43% had preceding angina	Increased in-hospital mortality (16% vs. 12%)
Bogaty et al. (1993) [14]	Retrospective cohort	102 (First AMI)	Prior stable angina	54% had an unheralded presentation	Unheralded group had less extensive disease (1.3 vs. 2.1 vessels)
Cupples et al. (1993) [35]	Prospective population-based cohort	828 (AMI)	Pre-existing cardiovascular conditions (angina, intermittent claudication, cerebrovascular disease, congestive heart failure)	68.6% of men and 60.5% of women had no pre-existing cardiovascular conditions	All-cause mortality at 34 years did not significantly differ between both groups
Danchin (2018) [31]	Retrospective registry analysis (FAST-MI program)	13,130 (AMI)	Previous diagnosis of CAD	26% had known CAD	Patients with known CAD were older with higher GRACE scores
Fujita et al. (1987) [15]	Prospective observational study	37 (AMI)	Angina >1 week before event	49% had a history of angina	Pre-infarction angina strongly associated with development of collateral vessels (50% vs. 11%)
Han et al. (2020) [27]	Prospective registry analysis (KAMIR-NIH registry)	5167 (STEMI)	Previous angina at any time prior to AMI	22% had previous angina	Cumulative MACE rate lower in previous angina group (4.9% vs. 8.0%); similar multi-vessel disease
Harper et al. (1979) [16]	Prospective cohort	577 (AMI)	Chronic angina (>1 month before AMI)	31% had chronic angina	Chronic angina more likely to result in subendocardial vs. transmural infarction
Herlitz et al. (1993) [24]	Retrospective cohort	917 (AMI)	History of chronic angina	34% had chronic angina	Reported early protective effect of angina on prognosis (though this pooled chronic angina and angina of short duration)
Herrett et al. (2014) [33]	Prospective population-based cohort	16,439 (AMI)	Existing ischemic disease (coronary, cerebrovascular or peripheral vascular disease)	49% had known ischemic disease	Mortality in first 7 days higher for those with existing disease
Herrett et al. (2013) [34]	Prospective cohort	8264 (STEMI/NSTEMI)	Known atherosclerosis (angina, CAD, cerebrovascular disease, peripheral arterial disease)	41% had a prior diagnosis of atherosclerosis	STEMI more likely upon first presentation (71%) vs. NSTEMI (50%)
Jacquemin et al. (1997) [19]	Retrospective cohort	151 (AMI)	Chronic angina (>1 month before AMI)	34% had chronic angina	Prior angina independently related to higher follow-up mortality and in-hospital heart failure
Kloner et al. (1995) [17]	Prospective trial cohort (TIMI 4)	416 (STEMI)	History of previous angina	52.4% had previous angina	Lower in-hospital death and shock in those with previous angina
Kloner, Shook et al. (1998) [28]	Prospective trial cohort (TIMI 9B)	3002 (AMI)	History of previous angina and time of onset	20% had a history of angina >1 month before event	Rest of analysis focused on angina <24 h before event, so not applicable
Kobayashi et al. (1998) [18]	Prospective cohort	1637 (first AMI)	History of previous angina	63% had previous angina	Lower in-hospital mortality (6.9% vs. 11%)
Matsuda et al. (1984) [13]	Retrospective cohort	31 (AMI LAD occlusion)	Angina >1 week before event	48% had a history of angina	Better ejection fraction (47% vs. 35%) and fewer wall motion abnormalities in the angina group
Midwall et al. (1982) [23]	Retrospective cohort	87 (AMI)	History of previous angina	28% had a history of angina	No angina group had more 1-vessel disease (60% vs. 9%) and fewer collaterals (46% vs. 71%)
Manfroi et al. (2002) [12]	Cross-sectional	104 (AMI)	History of previous angina	51% had previous angina	Hypertension was more present in patients with angina prior to MI
Siudak et al. (2018) [1]	Retrospective registry analysis	123,965 (AMI)	Previous diagnosis of CAD	77% first-presentation CAD	First-presentation patients were younger and had more 1-vessel disease
Solomon et al. (2004) [21]	Prospective trial cohort (VALIANT)	283 (Anterior MI)	Chronic angina (>3 months before AMI)	39% had chronic angina	Angina group had lower peak CK and less LV remodeling at 90 days
Schmidt et al. (2015) [30]	Retrospective nation-wide registry	70,458 (First AMI)	Outpatient diagnosis of stable/unstable angina	18.4% had known angina	No significant difference in 30-day or 1–5-year mortality between groups
Pierard et al. (1988) [22]	Prospective cohort	732 (First AMI)	Chronic angina (>1 month before AMI)	61% had preceding angina	Non-significant difference in ejection fraction function and peak CK
Romero-Farina et al. (2008) [29]	Retrospective	131 (AMI)	Chronic angina prior to AMI	21% had chronic angina	Chronic angina group had better EF and higher myocardial viability
Roubin et al. (1983) [25]	Prospective	229 (AMI)	First symptomatic presentation of CAD	60% first symptomatic presentation was AMI	Most first-presentation patients had 1-vessel disease

Abbreviations: AMI: acute myocardial infarction; CAD: coronary artery disease; EF: ejection fraction; MACE: major adverse cardiovascular events; STEMI: ST-elevation myocardial infarction; CK: creatine kinase.

### 3.2. Sex-Based Differences in the Presence of CAD Prior to AMI

Sex-based differences in AMI with or without prior CAD were inconsistently reported across studies. Most found that sex did not affect whether patients with AMI had known CAD [12,13,16,18,28,29]. However, Behar et al. found that angina prior to MI was more common in women (49%) compared to men (41%) (*p* < 0.001) [11]. Other studies showed similar findings [21,33]. Similarly, Pierard et al. reported a higher proportion of women in patients who experience angina prior to MI compared to those who do not (22% vs. 12%, *p* < 0.001) [22]. In Siudak et al.’s Polish registry study, women were more likely to present with AMI as their first CAD event (34% vs. 30%, *p* < 0.001) [1,2].

### 3.3. Young Age as a Risk Factor

Across multiple studies, patients with AMI as the first manifestation of CAD tended to be younger. Danchin et al. reported a mean age was 64 in the first AMI without known CAD group vs. 71 years in those with known CAD (*p* < 0.001) [31,32]. Siudak et al. reported a similar trend; 66 in the first AMI without known CAD group vs. 69 in those with known CAD (*p* < 0.01) [1,2]. Patients with a history of angina were also found to be younger than those without [11,14,16,22,23,30]. However, some studies found no significant age difference between the two groups [13,16,22,29]. Nonetheless, the majority of the higher-quality, more-recent evidence suggests that patients who present with AMI as their first manifestation of CAD tend to be younger.

### 3.4. The Role of Diabetes Mellitus

Behar et al. found that angina pectoris prior to AMI was more common in diabetic patients: 49% of patients with prior angina had diabetes, compared to 42% of those without prior angina (*p* < 0.0001) [11]. Similarly, Bogaty et al. reported a higher prevalence of diabetes in patients with stable angina and no history of acute events compared to those with unheralded AMI (i.e., no prior CAD) (*p* = 0.02) [14]. Similar findings were reported in the study by Schmidt et al. [30]. Siudak et al. found a similar pattern in their large PCI-treated AMI cohort: diabetes was more common in the group with known CAD (30%) compared to those without prior CAD (19%) [1,2]. Interestingly, eight studies found no significant difference in diabetes prevalence between the two groups [12,17,18,19,20,21,23,28,29].

### 3.5. Smoking as a Risk Factor

Most studies found no significant difference in smoking prevalence between patients presenting with AMI as their first CAD manifestation versus those with known CAD [12,17,18,23,28,29]. However, the Polish registry study reported a higher prevalence of smoking in patients with AMI as their first CAD event: 28% were smokers compared to 20% in the group with known CAD (*p* < 0.01) [1,2]. Similarly, two smaller studies observed the same trend, with a higher proportion of smokers in the unheralded AMI group [14,22].

### 3.6. Hypertension and Dyslipidemia

The majority of the studies that included hypertension in their analysis found no significant differences between the two groups [17,18,23,29]. However, Siudak et al. found that hypertension was more common in patients with AMI and previously diagnosed CAD (77%) compared to those with undiagnosed CAD prior to MI (39%, *p* = 0.006) [1,2]. Other studies also reported hypertension to be more frequent in patients with a history of angina [12,28,30]. Dyslipidemia and cholesterol data were reported less frequently. However, among the studies that did include these variables, most did not find significant differences in cholesterol levels between patients with established CAD and those without [12,18,23,29]. Jaquemin et al. found dyslipidemia to be more common in patients with a history of angina [19].

### 3.7. Higher Peak Cardiac Enzymes in Unheralded AMI

Patients with AMI as the first manifestation of CAD tend to have higher peak cardiac enzyme levels compared to those with prior CAD. Solomon et al. compared maximum creatine kinase (CK) levels and found they were significantly higher in patients with no prior angina (2701)compared to those with prior angina (2119] (*p* = 0.016) [21]. Similarly, Herrett et al. reported that peak troponin levels were significantly higher in patients with AMI as the first manifestation of CAD (2.6 ug/L) vs. those with existing CAD (1.4 ug/L, *p* < 0.001) [33]. These findings are consistent with several other studies [11,17,20,28].

### 3.8. Left Ventricular Ejection Fraction After AMI

Patients with a history of angina generally had better left-ventricular ejection fractions (LVEFs) following MI compared to those without prior angina. In Matsuda et al.’s study of 31 patients, the mean LVEF was significantly higher in patients with a history of angina (47%) compared to those without (3%, *p* < 0.005) [13]. Similarly, Romero et al. analyzed 131 patients and found that the average LVEF was higher in those with prior angina (34%) versus those without (31%, *p* = 0.019) [29]. However, it is worth noting that this study only included patients with reduced LVEFs (<40%). In contrast, a few studies found no significant differences in LVEFs between patients with and without prior angina [21].

### 3.9. Angiographic Findings

Bogaty et al. performed angiographic evaluation within 3 months of an “unheralded” AMI (defined as AMI occurring in patients without prior evidence of CAD) and within 2 years for patients with stable angina and no history of acute events. The authors observed a significantly lower burden of coronary artery disease in the AMI group, with an average of 1.3 diseased vessels compared to 2.1 vessels in the stable angina cohort (*p* < 0.001) [14]. In a smaller cohort, Fujita et al. investigated 37 patients with AMI who underwent intracoronary thrombolytic treatment within 6 h of symptom onset. The study compared patients with and without pre-infarction angina, defined as chest pain occurring >1 week before the index event. While the number of diseased vessels did not differ between the groups, collateral circulation was significantly more prevalent in patients with pre-infarction angina (50% vs. 11%, *p* < 0.05) [15]. Han et al. also found similar levels of multi-vessel disease between both cohorts [27].

The recent Polish registry study by Siudak et al., the largest cohort to date, corroborated prior findings. In this study, single-vessel disease was significantly more prevalent among patients with first-presentation AMI (46%) compared to those with known CAD prior to MI (30%, *p* < 0.001) [1,2], further supporting the association between fewer diseased vessels and AMI as the initial manifestation of CAD. Consistent with these findings, Midwall et al. reported that single-vessel disease was significantly more common among patients without prior angina: 60% of patients without a history of angina had single-vessel disease vs. 8.8% of those with prior angina (<0.005). Conversely, three-vessel disease was observed more frequently in patients with prior angina (38% vs. 11%, *p* < 0.005). Patients with pre-existing angina were also significantly more likely to exhibit collateral formation [23]. In line with this observation, Harper et al. demonstrated that patients with a history of chronic angina preceding AMI were more likely to develop subendocardial infarction, whereas patients without prior angina were more prone to transmural infarction [16]. However, not all studies confirmed these associations. Kloner et al. reported that although patients with AMI and a history of angina were more likely to have multi-vessel disease (68% vs. 55% *p* = 0.01), they did not demonstrate a significant difference in collateral formation [17].

### 3.10. Mortality and Major Adverse Cardiac Events (MACE)

Behar et al. reported significantly higher 5-year mortality among patients with a history of angina pectoris prior to AMI compared to those without (26% vs. 19%). Patients with prior angina also experienced worse in-hospital outcomes, including a higher incidence of cardiogenic shock, cardiac arrest, and overall mortality [11]. Similar findings were observed by Pierard et al., where prior angina was associated with worse short-term outcomes [22]. Herrett et al. found that mortality in the first 7 days of hospitalization was higher in patients with established ASCVD [33]. However, some studies found no statistically significant differences in short- or long-term mortality between patients with and without prior angina [31,32], including 800 patients in the Framingham cohort followed for over 30 years [35]. In contrast, other studies demonstrated improved survival in patients with known CAD prior to AMI. Kloner et al. and Kobayashi et al. both reported that in-hospital mortality was significantly higher in patients without prior angina [18,28].

## 4. Discussion

The reported incidence of AMI as the first presentation of CAD varies across studies. However, the majority of studies demonstrate that at least 50% of AMI occurs in patients without prior symptoms or known CAD. The discrepancy in incidence across studies can be attributed to differences in the study design, diagnostic tools available, and importantly, the definition of key variables. For instance, there are inconsistencies in the definition of AMI itself: some studies include only ST-elevation myocardial infarction (STEMI), others only include anterior STEMIs, and some include both STEMI and non-ST myocardial infarction (NSTEMI). This distinction is critical to interpreting the results of these studies. Recent evidence has shown that while STEMI patients are at a higher mortality risk acutely, NSTEMI patients may face a higher risk of recurrent events due to more extensive underlying atherosclerotic burden [36]. Perhaps the variation in disease burden between NSTEMI and STEMI affects the proportion considered as an inaugural presentation, as evidenced by the work of Herrett–George et al. [34].

Interestingly, despite advances in the detection of subclinical CAD, the proportion of patients presenting with AMI as the first manifestation of CAD does not seem to be decreasing. However, this observation must be interpreted with caution due to significant secular bias. The transition from CK-MD to high-sensitivity troponin assays has allowed for a lower threshold of diagnosis for AMI, making it difficult to compare studies decades apart. Moreover, the shift from symptom-based cohorts (angina vs. no angina) to modern diagnosis-based cohorts (history or no history of CAD or ASCVD) limits the comparability of these studies. Thus, it is difficult to interpret whether there is a true epidemiological shift. However, with advances in preventive cardiology, patients with “known CAD” are more likely to benefit from evidence-based secondary prevention measures, such as statins, antiplatelet agents, and aggressive comorbidity management, therefore reducing the risk of progressing to myocardial infarction. In contrast, those with undiagnosed CAD would not receive lifestyle and pharmacological preventative care and may be more likely to experience AMI. As a result, it is possible that the group of patients who go on to develop AMI may be increasingly composed of those without a prior diagnosis of CAD.

This hypothesis highlights the urgent need to identify this group of individuals who remain undiagnosed until their first coronary event. These individuals may not be identified by existing screening guidelines, and their risk factors may differ from those with typical anginal symptoms. Identifying their characteristics and risk factors could modify screening strategies for CAD and potentially reduce the burden of AMI as a first manifestation of coronary artery disease.

Our review revealed a lack of consensus across studies on whether sex significantly influences the likelihood of AMI as the first clinical manifestation of CAD. Of note, many studies did not include a significant number of women, highlighting a gap in the literature. Future studies should be adequately powered to evaluate sex as an independent risk factor. Sex is an important risk factor that must be better understood. Women often present with vague and non-specific symptoms, sometimes referred to as “non-cardiac” symptoms of heart disease. These include fatigue, dyspnea, and weakness, and differ from the typical exertional chest pain more commonly reported in men [37]. These “atypical” presentations may contribute to diagnostic delays and an under-recognition of CAD in women. Moreover, emerging evidence suggests that certain risk factors for CAD may differ between men and women. Notably, female-specific risk factors such as pregnancy-related issues (i.e., preeclampsia, gestational diabetes, etc.), polycystic ovary syndrome, auto-immune diseases and premature menopause increase the risks of CVD and atherosclerosis. The potential cardioprotective role of estrogen has been studied, though controversies remain [38]. Taken together, these findings suggest that risk factors and symptoms/manifestations of CAD may be influenced by sex. This diagnostic gap could explain, at least in part, why some women experience AMI as their first recognized manifestation of CAD; however, further research is needed to clarify this phenomenon.

Interestingly, several studies in our review found that diabetes was more prevalent among patients with prior angina or known CAD, rather than in those presenting with AMI as the first manifestation of coronary artery disease. This appears somewhat counterintuitive, as one would expect diabetic patients to be in the “no angina” group due to what is commonly referred to as “silent ischemia”. Indeed, it is thought that autonomic neuropathy in patients with diabetes mellitus leads to sensory denervation and abnormalities in pain perception. As a result, patients with diabetes tend to be more asymptomatic or have non-cardiac/atypical symptoms [39]. On the other hand, patients with diabetes are often followed more closely by a healthcare professional. This may lead to increased screening for CAD due to their elevated cardiovascular risk profile. In this way, diabetes acts as a “red flag” that prompts physicians to implement guidelines for primary prevention and cardiovascular disease risk stratification. Second, given that physicians know that diabetes is associated with autonomic dysfunction, they may be more inclined to investigate non-cardiac symptoms in diabetic patients. Consequently, these patients are more likely to be categorized into the “prior CAD or “prior angina” group rather than the “silent” or “first presentation” AMI group.

Elevated LDL levels were identified as a risk factor for AMI being the first manifestation of CAD. This likely reflects the fact that patients with stable angina or known CAD are more likely to be prescribed statins as part of guideline-directed medical therapy, which may result in lower LDL levels at baseline. In contrast, individuals without a prior diagnosis may have untreated dyslipidemia, contributing to their risk of presenting with unheralded AMI.

This review identified younger age as a risk factor for presenting with AMI as a first manifestation of CAD. This finding is particularly concerning, as it suggests that younger patients are more likely to present with a more severe and potentially fatal form of coronary artery disease without prior warning signs. It highlights the importance of identifying the specific risk factors associated with unheralded AMI in this population, and raises the question of whether earlier screening should be considered for individuals with these risk profiles. This finding becomes even more important considering that younger patients not only commonly present with unheralded AMIs, but these AMIs tend to be more fatal and cause larger myocardial damage compared to those in patients with known coronary disease. A leading hypothesis is that chronic ischemia associated with angina promotes the development of collateral circulation, which may help preserve myocardial perfusion during an acute plaque rupture and thereby reduce the infarct size [15].

While the studies in this review did not look at family history as a predictor of unheralded AMI, genetic factors have been shown to have the greatest impact on the early onset of cardiovascular events [40]. Therefore, future studies should consider integrating family history and possibly genetic profiling to better understand the underlying susceptibility of this population.

The relationship between traditional cardiovascular risk factors and the likelihood of unheralded AMI remains inconsistent across the literature. Given the serious implications of undiagnosed CAD, further research is essential to uncover the specific risk profiles of this vulnerable population. It is possible that the difference lies not only in patient-level characteristics but also in plaque-level characteristics, more specifically plaque burden, which can be measured by CCTA [41]. The COMBINE OCT-FFR trial, which consisted of patients with diabetes, demonstrated that it was not the presence of ischemia but rather the presence of intermediate stenosis and thin cap fibroatheroma (TCFA)-positive lesions that predicted future cardiac events [42]. The PECTUS Obs trial, which was performed in patients presenting with AMI, corroborated these findings [43]. Altogether the findings of these two trials, as well as the CILMA trial, suggest that plaque burden and plaque composition might be stronger predictors than ischemia [44]. Ischemia, and as a consequence ischemic symptoms, can occur during plaque progression; however, as also shown in the 5-year follow-up to the FAME 2 trial, an ischemia revascularization approach could not identify lesions that progress to future spontaneous MI [45]. Perhaps plaque characteristics, particularly vulnerability markers such as TCFA, rather than ischemia alone, will help predict future events [46]. Indeed, CCTA is evolving as an interesting screening tool for coronary disease detection. Moreover, new CCTA-derived and AI-derived vulnerability assessment might open the way to the early detection of high-risk coronary disease and implementation of tailored therapies, both medical and interventional, which could potentially lead to a reduction in future AMI risk [47].

A key limitation of our study is the nature of the available literature and its significant heterogeneity in study design, clinical era and key definitions of “prior angina” or “history of CAD”. For this reason, a scoping review, instead of a meta-analysis, was conducted. It allowed for a narrative review of the evidence while acknowledging that a single pooled prevalence would be inappropriate.

## 5. Conclusions

The current available literature shows that a significant proportion of patients presenting with AMI have no prior anginal symptoms or known CAD. A possible explanation for this finding is that the current guidelines reserve coronary anatomical assessment, and therefore more active medical prevention and/or interventional treatments, to symptomatic patients. Progressively increasing the use of CCTA as a new screening tool may enhance the early detection and treatment of high-risk but asymptomatic patients and consequently reduce the incidence of future MI events.

## Data Availability

No new data were created or analyzed in this study.

## Abbreviations

The following abbreviations are used in this manuscript:

CAD	Coronary artery disease
AMI	Acute myocardial infarction
MACE	Major adverse cardiac events
STEMI	ST-elevation myocardial infarction
NSTEMI	Non-ST-elevation myocardial infarction
TCFA	Thin-cap fibroatheroma
CCTA	Coronary computed tomography angiography
